# Supplementary figures and images for: Genetically regulated gene expression underlies lipid traits in Hispanic cohorts
Source: PLoS One. 2019 Aug 8;14(8):e0220827. doi: 10.1371/journal.pone.0220827 (PMC6687110; doi:10.1371/journal.pone.0220827)

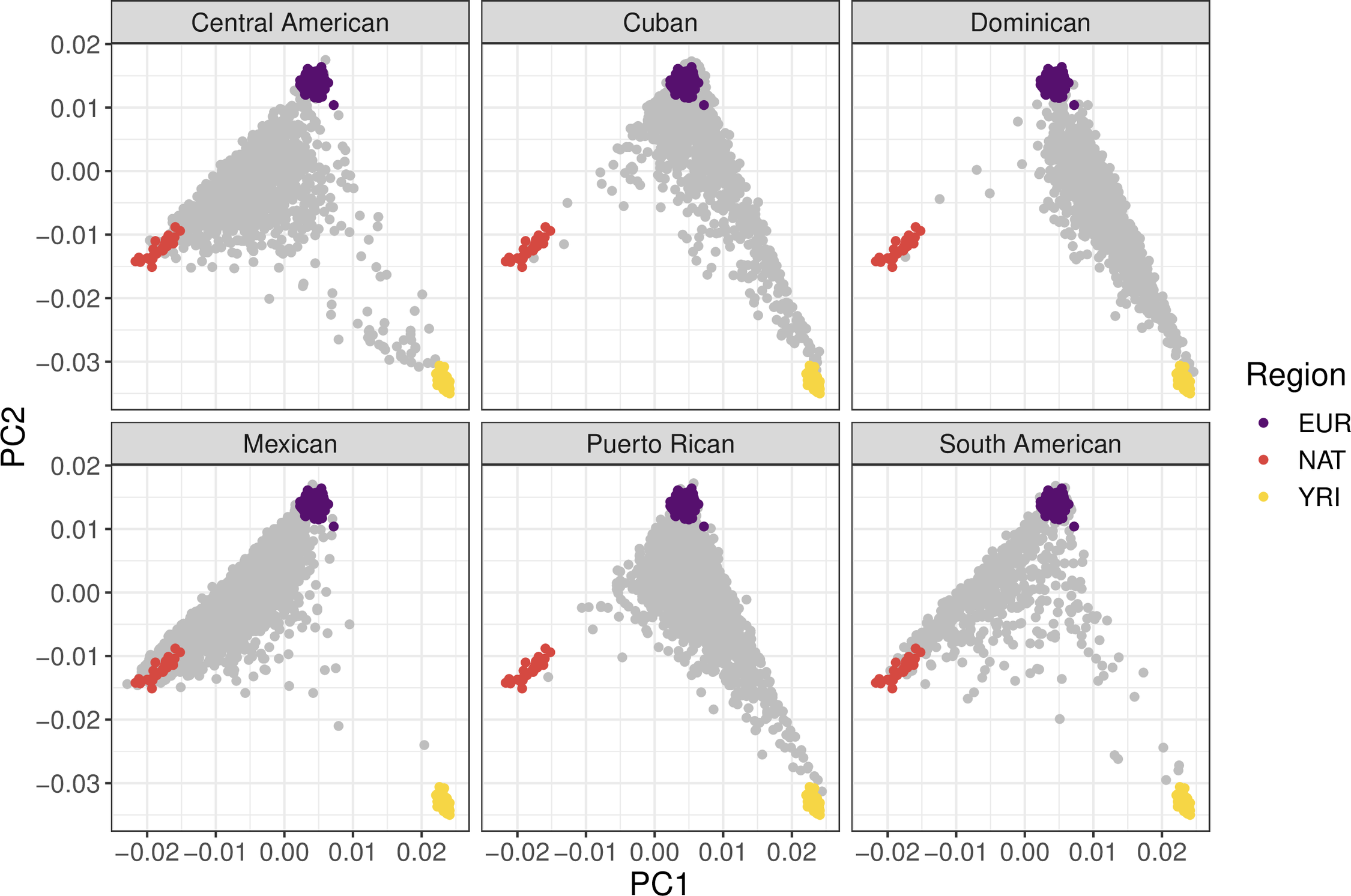

Supplement: S1 Fig — PC1 vs. PC2 is plotted for each individual separated by their self-identified region. From previous observations and studies, Hispanic populations have multiple continental ancestries due to a previous history of colonization and slavery: African (bottom right, YRI), Native American (left, NAT), and European (top, EUR). Caribbean populations, such as the Cuban, Dominican, and Puerto Rican groups, tend to be mainly admixed between African and European, while mainland populations such as Mexican, Central American, and South American, tend to be mainly admixed between Native American and European. (TIF) [file pone.0220827.s003.tif]

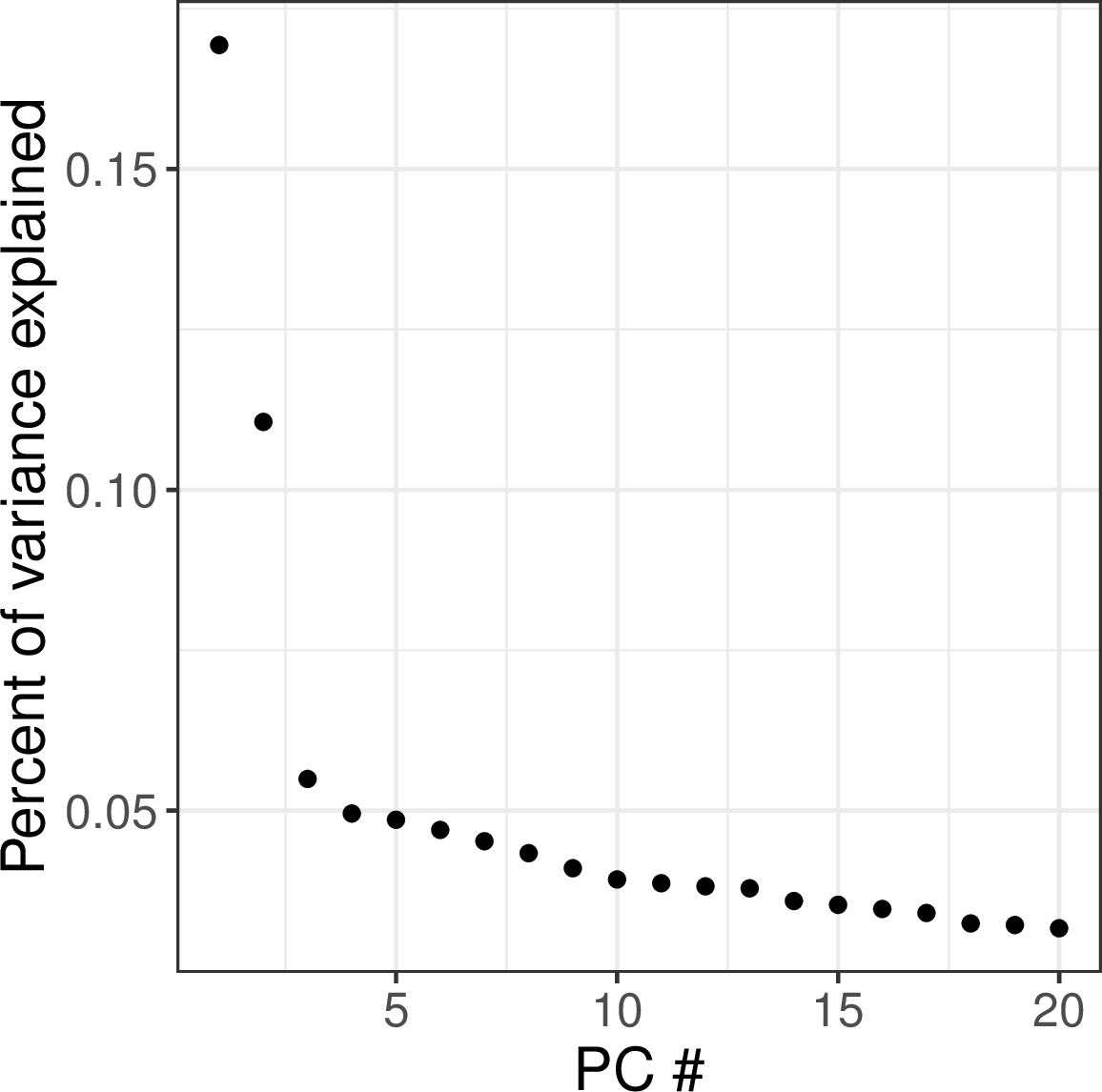

Supplement: S2 Fig — Principal components 1, 2, and 3, explain 16.934%, 11.060%, and 5.494% of the variance, respectively. All other principal components explain < 5% of the variance each. All analyses used 5 PCs as fixed effects, as previously used in analyses of HCHS/SoL. (TIF) [file pone.0220827.s004.tif]

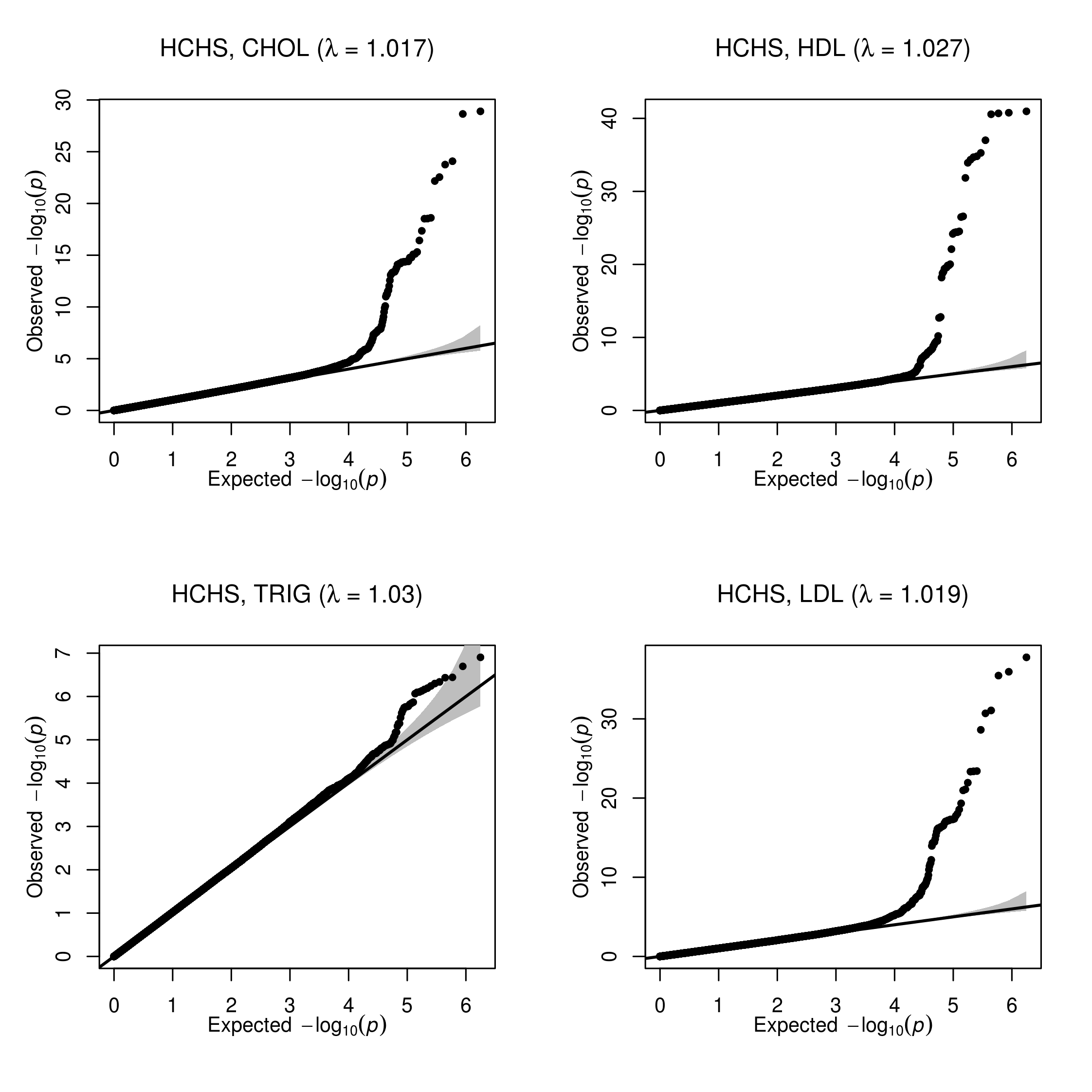

Supplement: S3 Fig — Genomic control lambda values (λ) indicate little genome-wide deviation from the significance expectation line in any of the GWAS results. (TIF) [file pone.0220827.s005.tif]

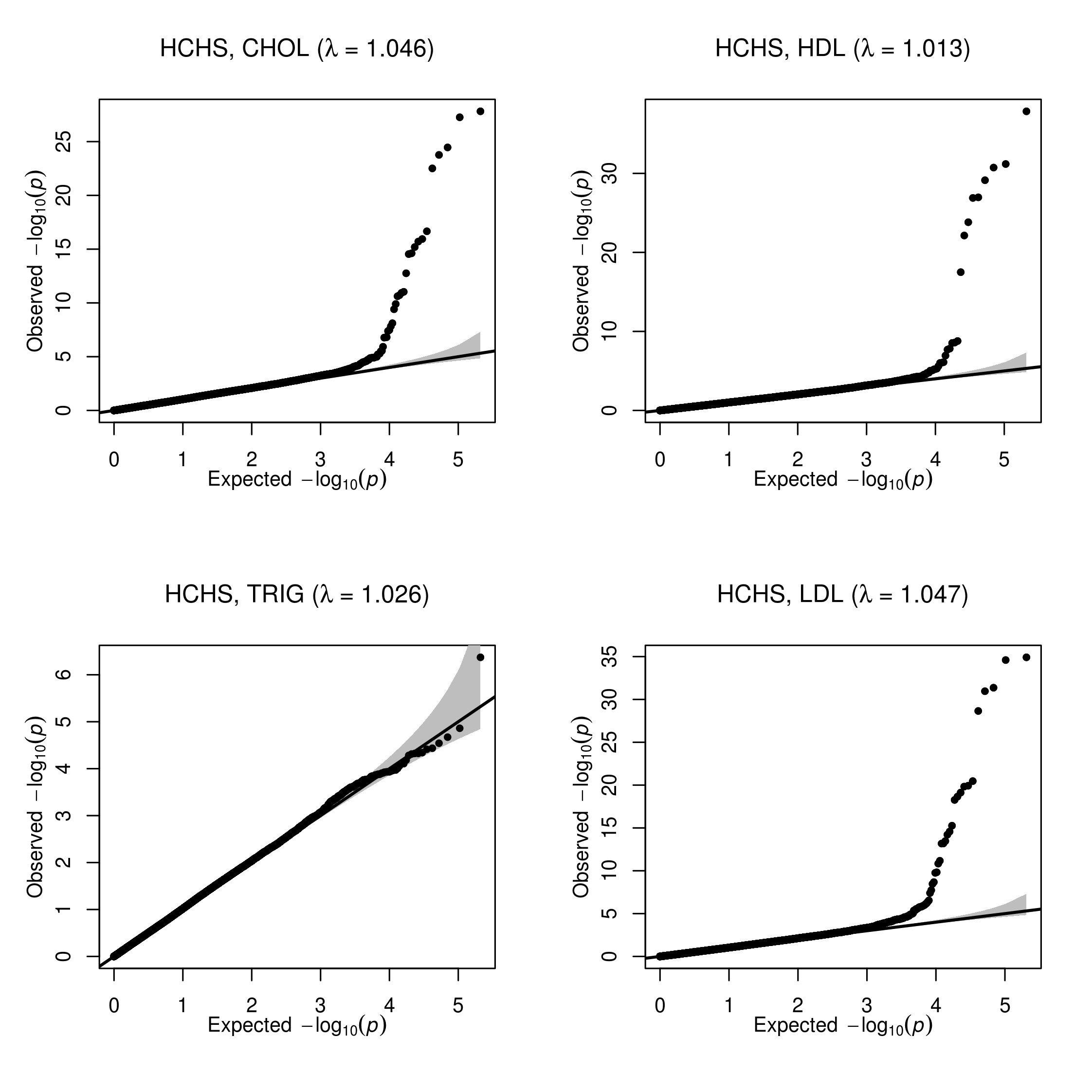

Supplement: S4 Fig — PrediXcan results for all 44 GTEx tissue models and 5 MESA monocyte populations are combined. Each point is a gene-tissue or gene-population association. Genomic control lambda values (λ) indicate little genome-wide deviation from the significance expectation line in any of the PrediXcan results. (TIF) [file pone.0220827.s006.tif]
